# First Insight into Nutraceutical Properties of Local Salento *Cichorium intybus* Varieties: NMR-Based Metabolomic Approach

**DOI:** 10.3390/ijerph18084057

**Published:** 2021-04-12

**Authors:** Chiara Roberta Girelli, Francesca Serio, Rita Accogli, Federica Angilè, Antonella De Donno, Francesco Paolo Fanizzi

**Affiliations:** Department of Biological and Environmental Sciences and Technologies, University of Salento, Prov.le Lecce-Monteroni, 73100 Lecce, Italy; chiara.girelli@unisalento.it (C.R.G.); francesca.serio@unisalento.it (F.S.); rita.accogli@unisalento.it (R.A.); federica.angile@unisalento.it (F.A.); antonella.dedonno@unisalento.it (A.D.D.)

**Keywords:** nutraceuticals, metabolomics, food safety, human health, NMR-spectroscopy

## Abstract

Background: Plants of genus *Cichorium* are known for their therapeutic and nutraceutical properties determined by a wealth of phytochemical substances contained in the whole plant. The aim of this paper was to characterize the metabolic profiles of local Salento chicory (*Cichorium intybus* L.) varieties (“Bianca”, “Galatina”, “Leccese”, and “Otranto”) in order to describe their metabolites composition together with possible bioactivity and health beneficial properties. Methods: The investigation was performed by ^1^H-NMR spectroscopy and Multivariate Analysis (MVA), by which the metabolic profiles of the samples were easily obtained and compared. Results: The supervised Partial Least Squares Discriminant Analysis (PLS-DA) analysis showed as “Bianca” and “Galatina” samples grouped together separated by “Leccese” and “Otranto” varieties. A different content of free amino acids and organic acids was observed among the varieties. In particular a high content of cichoric and monocaffeoyl tartaric acid was observed for the “Leccese” variety. The presence of secondary metabolites adds significant interest in the investigation of *Cichorium inthybus*, as this vegetable may benefit human health when incorporated into the diet. Conclusions: The ^1^H-NMR (Nuclear Magnetic Resonance Spectroscopy) based characterization of Salento chicory varieties allowed us to determine the potential usefulness and nutraceutical properties of the product, also providing a method to guarantee its authenticity on a molecular scale.

## 1. Introduction

To date, several organizations such as the World Health Organization (WHO) and Food and Agriculture Organization (FAO) promote increasing intake of vegetables in human nutrition [1], with important ethical, environmental, and reduced costs advantages [2]. Much of their preventive activity is thought to be provided by phytochemicals capable of antioxidant, antimutagenic, cytotoxic, antifungal, and antiviral activities. Notwithstanding a large amount of studies on this topic, nutrient composition of vegetables is very difficult to evaluate. Indeed, each vegetable variety is characterized by a particular chemical composition (dietary fiber, phenolic compounds, flavonoids, oils, plant sterols, proteins, prebiotics, probiotics, anthocyanins, carotenoids, and many others) [3] influenced by several factors (genetic, environment, transportation, and storage conditions). Apulia region (southern Italy) is particularly rich in agro-biodiversity, in traditional food products with high nutraceutical and organoleptic values. Therefore, their characterization is highly advisable. Recently, the Apulia Region planned specific actions under the 2014–2020 Rural Development Programme (RDP) to preserve regional genetic biodiversity. In particular, this paper is related to the project “Biodiversity of the Puglia’s vegetable crops (BiodiverSO)”, one of the five integrated project funded by Puglia Region Administration under RDP [4,5]. Among these products, chicory (*Cichorium intybus* L., 1753) is a perennial herbaceous plant of the Asteraceae family, native of Mediterranean and middle Asia regions [6] and widely cultivated in most temperate areas such as North America, parts of Asia and Europe [7,8,9]. South Italy, with particular reference to the Salento area (Lecce and Brindisi Apulia provinces) is one of the major chicory cultivations areas.

*Cichorium intybus* can be divided according to the purpose and use for which it was cultivated [10]: “root” chicory, predominantly used for inulin extraction; “forage” chicory, to intensify herbage obtainability in perennial pastures for livestock; “Witloof Chicory” or “Belgian Endive”, commonly grown as an industrial crop; the variety “leaf” chicory indicates different kinds of crops with colors ranging from white to red [7]. The “Radicchio,” “Sugarloaf”, and “Catalogna” sub-groups are “leaf” chicory varieties cultivated in Italy for both leaves and stems. All type of chicories are characterized by the presence of a large amount of phytochemicals with potential nutraceutical effects, such as phenolic acids, flavonoids, and water-soluble anthocyanin pigments, responsible for the color of red chicory [7,11]

These vegetables can be used raw for the preparation of salads or cooked for the realization of first courses and/or side dishes. Among the various types, different in biological and morphological characteristics, the “Catalogna” group includes various categories that have a “head” integrated by numerous sprouts. They are similar in appearance to the asparagus shoots which results almost everywhere also in the denomination of “asparagus chicory” [8,12].

Within the Apulian territory they are known with different local names, such as “Catalogna puntarelle”, “Brindisina”, “Bianca”, “Galatina”, “Pugliese”, “Leccese”, “Otrantina”, and “Molfettese” [13]. Thanks to the high content in bioactive compounds, important health properties have been attributed to the genus *Cichorium*: hepatoprotective, anti-inflammatory, antioxidant, sedative, immunological, reproductive, gastro-protective, antidiabetic, and analgesic [14]. Several studies reported the chemical composition and health benefits of chicory [15]. It was considered as a source of inulin, oligofructose and sesquiterpene lactones, caffeic acid derivatives (chicoric acid, chlorogenic acid, isochlorogenic acid, and dicaffeoyl tartaric acid) with benefic effects on health [14,16,17,18,19]. Chicory has a nutritional quality comparable to lucerne as it contains similar proportions of protein, lipid, minerals, and other nutrients [9]. In particular, anthocyanins are widely distributed phenolics in chicory and exhibit significant health-beneficial properties due to their antioxidant and anti-inflammatory activities [14,16,17,18]. The awareness towards food safety and quality has gained significant importance among consumers in recent years, as they are interested in authentic foods and drinks that provide healthy nutrients and bioactive compounds. Therefore, it becomes necessary to adopt high standards in food quality also by the use of analytical methods in order to detect the composition and the physico-chemical properties of food matrices. This need has led to the development of analytical approaches for a comprehensive assessment of the benefits and risks associated with food intake [20]. For this purpose, metabolomics quickly emerged as an effective tool in food and nutrition sciences for products characterization [21,22]. This approach could encourage the development and production of safe and high-quality food also promoting nutrition-based human health. Metabolomics uses an untargeted approach that takes into account the most abundant low-molecular weight compounds present in any biological matrix. Metabolomics could be defined as the systematic study of the chemical fingerprints originated by specific cellular processes. Among the analytical techniques used, Nuclear Magnetic Resonance Spectroscopy (NMR), together with Chromatography coupled Mass Spectroscopy (HPLC-MS) have been extensively used in the metabolomics applied to foodstuffs studies. NMR spectroscopy is characterized by the ability of offering a wide range of information on metabolites within a single experiment, without components separation and with minimal requirement for sample preparation and pretreatment [23]. Original limitations of this technique due to the intrinsic low sensibility have been nowadays overcome thanks to high field instruments, cryoprobe assisted detection and high performance acquisition pulse programs [24]. NMR spectroscopy therefore results a high throughput, non-invasive, high reproducible and quantitative technique, that provides detailed information on molecular basis of complex mixtures [25]. NMR-based metabolomics has shown great success in assessing the health and safety aspects of food and food processes, as well as providing valuable information on the quality status and authenticity of food products [22,26]. Plant metabolomics is a growing discipline successfully employed in plant functional genomics, food science, and human nutrition [27].

In this work, a NMR-based metabolomic approach was applied to four local varieties of chicory with the aim of improving the knowledge on their nutraceutical value; by identifying the bioactive compounds present, and therefore of the possible benefits for human health related to its consumption.

## 2. Materials and Methods

### 2.1. Leaf Samples Collection 

**Figure 1:**
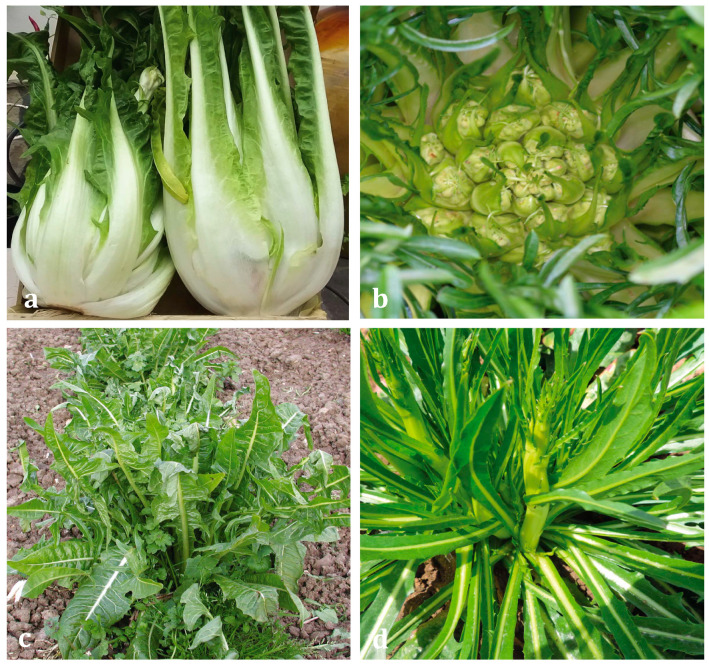
Chicory local varieties described in this paper. (**a**) “Bianca”; (**b**) “Galatina”; (**c**) “Leccese”; (**d**) “Otranto”.

A total of 36 chicory samples (edible leaves and stems) were obtained from 4 chicory local Salento varieties, “Bianca”, “Galatina”, “Leccese”, and “Otranto” (Figure 1). The local varieties studied are described in the database of National Register of Agricultural Biodiversity, established by National Ministerial Decree n. 1862 of 18/01/2018 [28], with the aim of protecting the local genetic resources of agricultural interest of vegetable, animal or microbial origin that run the risk genetic extinction or erosion [5]. Morphological characterization for each variety can be found in the Supplementary Material (Table S1).

Chicory samples were supplied by local farms in the Salento peninsula (province of Lecce) within the framework of the project “Biodiversity of Apulian vegetable species” (Rural Development Programme, European Agricultural Fund for Rural Development, Reg. EC. No. 1698/2005) [29]. Chicory plants were growth according to local traditional agronomic practices. Three plants for each variety were harvested at maturity according to size, skin colour change and consistency. “Bianca” and “Galatina” varieties were collected between December and January, “Leccese” variety on May, and “Otranto” on August. Each samples consisted of three technical replicates.

### 2.2. Sample Preparation for ^1^H NMR Analysis

Samples were prepared according to the experimental procedure as reported in literature [25]. Briefly, lyophilized plant material (100 mg) was weighted into an autoclaved 2 mL Eppendorf tube. Thereafter, 0.75 mL of CD_3_OD and 0.75 mL of KH_2_PO_4_ buffer in D_2_O (pH 5.9) containing 0.05% *w*/*v* TSP-d4 (sodium salt of trimethylsilyl propionic acid) were added to each sample. The content of the eppendorf tubes was mixed thoroughly with a vortex mixer at room temperature for 1 min and then sonicated for 10 min at room temperature. Samples were spun down in a microcentrifuge at 17,000 *g* for 20 min at 4 °C; then, 700 μL of the supernatant were filled into a 5 mm NMR tube.

### 2.3. ^1^H-NMR Spectra Acquisition and Processing

All measurements were performed on a Bruker Avance III 600 Ascend NMR spectrometer (Bruker, Ettlingen, Germany), operating at 600.13 MHz for ^1^H observation, equipped with a TCI cryoprobe incorporating a z axis gradient coil and automatic tuning-matching (ATM). Experiments were acquired at 300 K in automation mode after loading individual samples by a Bruker Automatic Sample Changer, interfaced with the software IconNMR (Bruker). For each sample a 1D sequence with pre-saturation and composite pulse for selection (zgcppr Bruker standard pulse sequence) was acquired, with 16 transients, 16 dummy scans, 5 s relaxation delay, size of fid of 64K data points, a spectral width of 12,019.230 Hz (20.0276 ppm) and an acquisition time of 2.73 s. The resulting FIDs were multiplied by an exponential weighting function corresponding to a line broadening of 0.3 Hz before Fourier transformation, automated phasing and baseline correction. Metabolite identification were based on ^1^H and ^13^C assignment by 1D and 2D omo and heteronuclear experiments and by comparison with literature data [30,31,32]. NMR data processing was performed by using TopSpin 3.6.1 (Bruker, Biospin, Milano, Italy). All spectra were referenced to the TSP signal (0.00 ppm). ^1^H NMR spectra were converted to a suitable form for multivariate analysis by segmentation into small rectangular bins (buckets) and integration by Amix 3.9.15 (Analysis of Mixture, Bruker BioSpin GmbH, Rheinstetten, Germany) software. Bucketing was performed within 10.00–0.5 ppm region, excluding the residual non-deuterated water (4.9–4.7 ppm) and methanol (3.40–3.30 ppm) signals. In order to account for small pH or concentration signals shift, the width of each bucket was fixed to 0.04 ppm. The total sum normalization was then performed in order to minimize small differences due to metabolites concentration and/or experimental conditions among samples [33]. The Pareto scaling method, which is performed by dividing the mean-centered data by the square root of the standard deviation, was then applied to the bucket reduced NMR spectra (variables). The data table arisen from all aligned buckets row reduced spectra was used for further multivariate data analysis. Each bucket row depicts the entire NMR spectrum, with all the molecules present in the sample. Moreover, each bucket in a buckets row reduced spectrum is labeled with the value of the central chemical shift for its specific 0.04 ppm width. The buckets are the variables used as descriptors for each sample in chemometric analyses. 

### 2.4. Multivariate Statistical Analysis

Software Simca-P version 14 (Sartorius Stedim Biotech, Umeå, Sweden) was used to accomplish multivariate statistical analysis. In particular, PCA (Principal Component Analysis), PLS-DA (Partial Least Squares Discriminant Analysis) and OPLS-DA Orthogonal Partial Least Squares Discriminant Analyses) were performed. The commonly used unsupervised analysis (PCA) shows the systematic variation in a data matrix X formed by rows (the considered observations), and columns (the variables which describe the samples) [34,35]. PLS-DA is the most used supervised analysis for the discrimination between samples of identified classes with different characteristics [36]. The PLS-DA is performed to refine the separation between groups of observations, rotating the main components such that a maximum separation among classes is obtained [37]. OPLS–DA is a modification of the PLS–DA method which filters out variation not directly related to the focused discriminating response. This is accomplished by separating the portion of the variance useful for predictive purposes from the non-predictive variance (which is made orthogonal). OPLS-DA is especially suited for highlighting class discriminating variable in the two class problems [38]. The internal cross-validation default method (7-fold) and the permutation test (40 permutations), available on the SIMCA-P software, were used in order to validate the statistical models [39]. The quality of the obtained models was described by the R^2^ and Q^2^ parameters. These parameters show completely different behavior with the model complexity increase. The R^2^X, R^2^Y, and Q^2^, describing the total variation in X, the variation in the response variable Y and the predictive ability of the models, respectively, were calculated [40]. The relative change in discriminating metabolite content among the observed groups was evaluated by analyzing the mean values +/− standard deviation of selected bucket reduced distinctive unbiased NMR signals after spectra normalization (to the total spectrum excluding the residual water region) [41,42]. Results, were validated by Analysis of variance (one-way ANOVA) with Tukey’s honestly significant difference (HSD) post-hoc test by using MetaboAnalyst 5.0 software (Genome Canada, Ottawa, Canada) [43].

### 2.5. Chemicals

All chemical reagents for analysis were of analytical grade. Deuterium oxide (99.9 atom %D) containing 0.05% wt 3-(trimethylsilyl)propionic-2,2,3,3 d4 acid sodium salt (TSP), Potassium phosphate monobasic were purchased from Armar Chemicals (Döttingen, Switzerland). Methanol-d4 (99.9 atom %D), potassium phosphate monobasic were purchased from CARLO ERBA Reagents (Milano, Italia). 

## 3. Results and Discussion

### 3.1. Visual Inspection of ^1^H NMR Spectra

**Figure 2:**
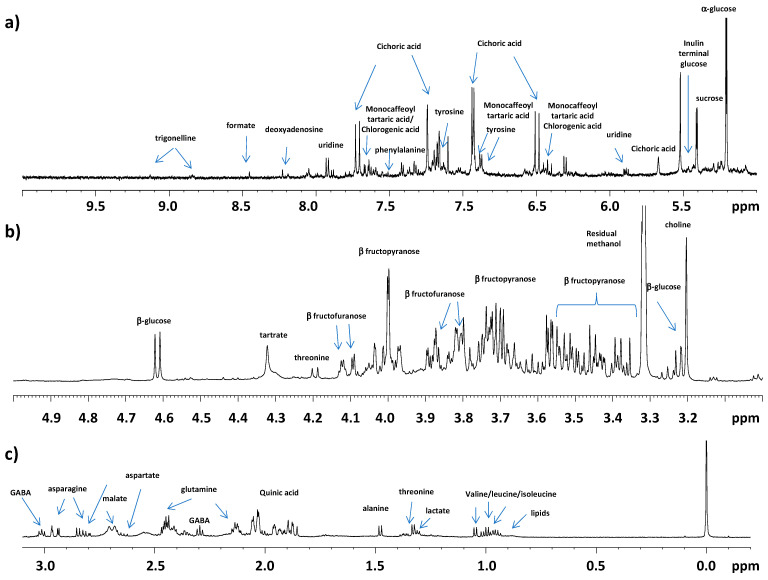
^1^H-NMR (Nuclear Magnetic Resonance Spectroscopy) typical spectrum of a *C. inthybus* aqueous extract samples. Expanded areas in of (**a**) (−0.5 to 3 ppm), aliphatic region; (**b**) (3–5 ppm) sugars region; (**c**) (5–10 ppm) aromatic region. The peaks of relevant metabolites are indicated.

Visual inspection of the 600 MHz ^1^H NMR typical spectra revealed a complex pattern of signals due to the presence of different classes of metabolites. In the low frequency field region of the spectra (0.5–3.00 ppm) the presence of amino acids, fatty acids chains from cell membrane [44], malic and quinic acid was assessed on the base of literature data [30,31] and bidimensional experiments. In particular, for the amino acids class, isoleucine, leucine, valine, threonine, alanine, glutamine, GABA (γ-amminobutyrate), and asparagine were identified (Figure 2a). As already known [14,15], chicory leaves are strongly rich in free amino acids. These organic molecules exhibit an important role for human health maintenance being recommended for a balanced and healthy diet. Overlapped with threonine doublet the signal of lactate was also identified. Malic and quinic acids were also observed in the spectra. As already reported [45] the presence of these organic acids was found in other vegetables belonging to *C. intybus.* The contribute to the organoleptic properties as well as the physiological role for malic [46] and quinic acid [45] were also described. Due to the presence of overlapping signals, the interpretation of the spectra in the 3.00–5.00 ppm (Figure 2b) was complex. Nevertheless, anomeric protons of *β* glucose, and *α* fructofuranose, *β* fructofuranose, and fructopyranose signals were observed. The detection of *β* fructofuranose signals could be also ascribable to inulin fructofuranose units [32]. Due to the intense overlapping region in ^1^H NMR spectrum, the 2D NMR ^1^H-^13^C hsqc NMR spectra were used in the assignments of these signals. Moreover, signals of choline and tartrate were also observed. Choline’s role in human health begins prenatally and extends into adulthood and old age [47,48]. Choline is an essential nutrient needed for human health, because of its importance for proper liver, muscle, and brain functions. It is also implicated in cellular membrane composition and lipid metabolism [48,49,50,51]. Humans can produce only small amounts of choline; therefore, this endogenously produced nutrient must be integrated through the diet to prevent deficiency. In the high frequency region of the spectra (5.0–9.5 ppm) signals ascribable to important phytochemicals could be observed (Figure 2c). In this region anomeric protons of other common sugars such as α glucose and sucrose were detected. The presence of a multiplet at 5.42 ppm about 0.02 ppm shifted with respect to the anomeric protons of sucrose was assigned to the anomeric proton of terminal glucose unit of inulin. This was confirmed by the assignments in the 2D ^1^H-^13^C hsqc spectrum (Figure S1). Inulin is constituted by a *β* fructofuranose polymer with a terminal glucose and represent the most abundant soluble polysaccharide in chicory roots [52]. Moreover, characteristic signals for the vinyl proton of caffeoyl moieties of hydroxycinnamates such as cichoric, and, partially overlapped, of monocaffeoyl tartaric and chlorogenic acids were identified on the basis of literature data [30,32] and 2D ^1^H-^13^C hsqc spectrum (Figure S1). Important anti-hepatotoxic activity of cichoric acid was already studied [53]. In addition, cichoric acid aqueous extract was described as antihyperglycemic agent [15]. The presence of other metabolites, such as trigonelline, formate, fumarate and nucleosides derivatives such as uridine and deoxyadenosine together with aromatic amino acid as phenylalanine and tyrosine were also identified as reported (Table 1 and Figure 2c).

The presence of metabolites with important nutritional value adds significant interest in the chemical characterization of *C. inthybus*. Moreover, the discrimination among local varieties could help to identify *C. inthybus* classes with specific biochemical features focusing on those particularly rich in healthy compounds.

### 3.2. Multivariate Statistical Analysis

**Figure 3:**
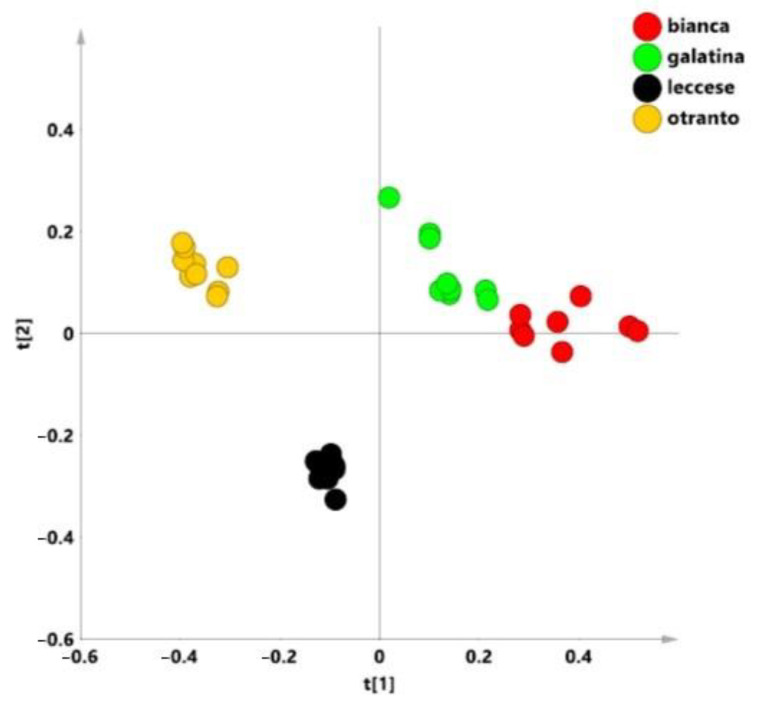
Principal Component Analysis (PCA) t[1]/[t2] scores plot for local *C. intybus* varieties t[1] and t[2] components explain 77.7% of the total variance.

In order to examine an overview of the whole bucket reduced NMR data set, a preliminary unsupervised PCA was applied. In this model, three components gave excellent descriptive parameters: R^2^X = 0.878 and Q^2^ = 0.833. The t1/t2 scores plot for the model (Figure 3), revealed a clear clustering of the samples according to the studied varieties. As observable from visual inspection of the scores plot, “Galatina” and “Bianca” varieties were placed as a macro group at positive values of t1 and t2 component. On the other hand, at negative values of t1 component, “Otranto” and “Leccese” samples were observed, clearly separated, and at negative and positive values respectively along the t2 component. The distance to the model of X (DMODX) for the model (Figure S2) indicated that no observations resulted outside the critical distance; therefore, all the samples were included in the model and used for further analysis. 

**Figure 4:**
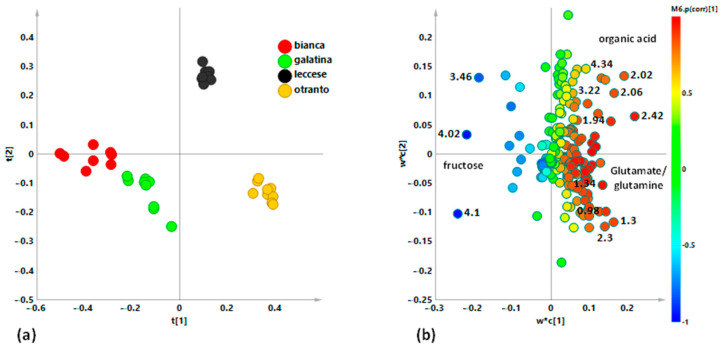
(**a**) Partial Least Squares Discriminant Analysis (PLS-DA) t[1]/t[2] scores plot for *C. inthybus* local varieties (three components, R^2^X (cum) = 0.873; R^2^Y (cum) = 0.934, Q^2^ (cum) = 0.92, p[CV]-anova = 0. (**b**) loading scatter plot for the PLS-DA model, colored according to the correlation scaled coefficient (* *p*(corr) ≥ |0.5|). The colour bar associated to the plot indicates the correlation of the metabolites in segregating among classes.

In order to refine the samples grouping observed in the unsupervised PCA model, a discriminant analysis was then performed. PLS-DA gave a very good model described by three components giving R^2^X = 0.873, R^2^Y = 0.934, and Q^2^ = 0.92. The excellent R^2^ and Q^2^ descriptive and predictive values parameters allowed a reliable further model interpretation. Studied samples were classified according to the considered varieties and, as observable from the scores plot, a clear group separation according to the first t1 component was obtained (Figure 4a). In particular, “Bianca” and “Galatina” class samples were observed, close enough between them, at negative values of the t1 component. “Otranto” and “Leccese” class samples resulted placed at positive values of the first principal component but clearly separated among the orthogonal component. The observed separation seemed to be attributed to the different harvesting period, winter and spring season for “Bianca”, “Galatina” and “Otranto”, and “Leccese” respectively [5]. Due to the more crunchy and tender texture of their shoots, “Bianca” and “Galatina” chicories are local varieties less resistant to cold than other Apulian local varieties of Catalonian chicory. These varieties do not tolerate cold and winter frosts but guarantee the product in winter season, being the long period of cultivation ensured by scalar transplants and scalar maturation. Their characteristics would allow them to be present on the “ready to use market”, with production and quality characteristics rather homogeneous throughout the availability period in the field. “Otranto” and “Leccese” instead, are varieties typical of the summer seasons. For the “Otranto” variety, a specific domestication, carried out by the farmers of the Otranto area was reported [5]. The aim was to adapt the autumn–winter crop of Catalonian chicory to the conditions of late spring and summer, thanks to availability of marshy places. On the other hand, the “Leccese” derives from a specific cultural phase shift of the “Galatina”, performed with the purpose of a May harvesting, when the local climatic conditions would be limiting for cultivation [5]. The loading scatter plot for the model (Figure 4b) was used to identify metabolites responsible for classes’ differentiation. Thus, buckets representing ^1^H-NMR signals at 3.36, 4.02, and 4.10 ppm were ascribable to NMR region diagnostic for fructose indicating a higher content of this sugar in “Bianca” and “Galatina” with respect to “Otranto” and “Leccese” class samples. Fructose is commonly used in the food industry as a sweetening substitute for sucrose [54] and seems to be the most abundant sugar present in chicory leaves followed by glucose and sucrose [52,55,56]. The “Bianca” variety has a sweet and aromatic flavor while “Galatina” is appreciated for the tenderness of the central heart which makes it particularly suitable for raw consumption. A great impact on the amount of carbohydrates is due to the considered chicory cultivar, but also to different planting and harvesting periods [56]. In particular, the harvest times was closely associated with the total sugar content, resulting greater in early November. The role of soluble sugars in enhancing cold tolerance was already described [57]. In particular, the higher relative content of fructose observed for “Galatina” and “Bianca” varieties is in accord with the increase in level of fructose sugars as important non-enzymatic antioxidative defense in plants under cold-related stress, as reported [58]. On the contrary, higher values of specific signals related to the NMR region ascribable to aliphatic amino acids as glutamate/glutamine, organic acids such as acetate and lactate were observed for “Otranto” and “Leccese” classes. Amino acids are organic compounds with an important role in human health maintenance [59]. Glutamate and glutamine, with aspartate, asparagine, glycine, serine, and arginine are the main amino acids in chicory [60,61].

**Figure 5:**
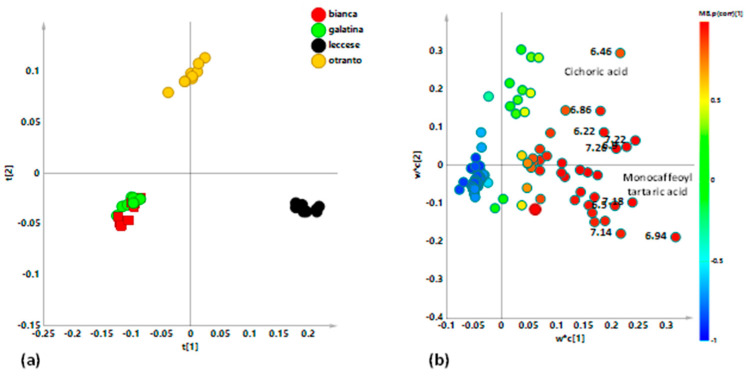
(**a**) PLS-DA t[1]/t[2] scores plot for *C. inthybus* local varieties (three components, R^2^X (cum) = 0.968, R^2^Y (cum) = 0.766, Q^2^ (cum) = 0.726, p[CV]-anova = 1.357 × 10^−18^) focusing aromatic spectral region. (**b**) loading scatter plot for the PLS-DA model, colored according to the correlation scaled coefficient (* *p*(corr) ≥ |0.5|). The colour bar associated to the plot indicates the correlation of the metabolites in segregating among classes.

**Figure 6:**
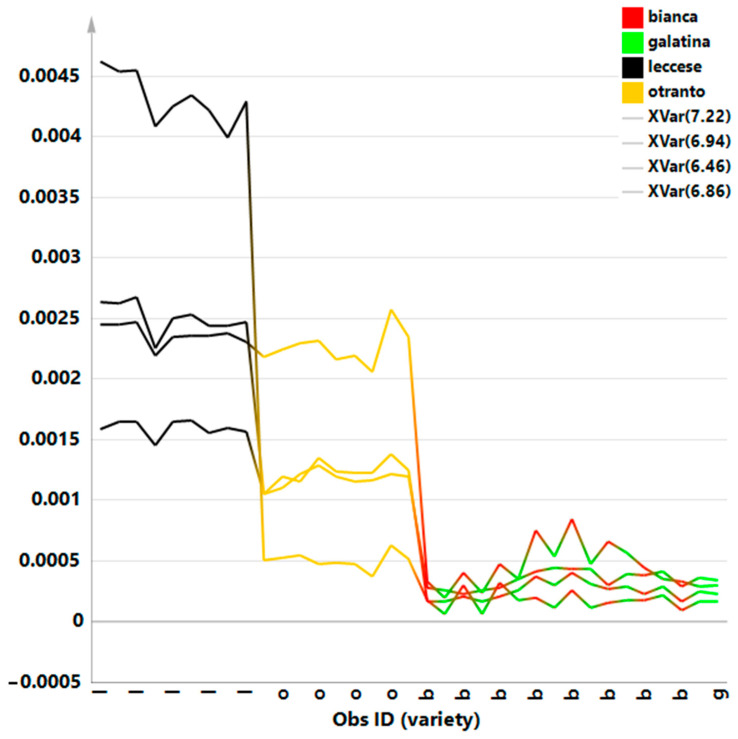
Line trend plot reporting the selected variables trend colored according to the varieties class.

Moreover, in order to thoroughly analyze the potential differences in the metabolites with distinctive signals in the aromatic region we performed a new bucketing from ^1^H zg spectra was further performed considering only the region between 9.00 and 6.00 ppm. Further discriminant PLS-DA analysis provided a model with good values for the R^2^ and Q^2^ parameters: three components gave R^2^X = 0.918; R^2^Y = 0.766 and Q^2^ = 0.726. Visual inspection of the scores plot (Figure 5a) showed as “Bianca” and “Galatina” class clustered together as a single group at negative values of the first component. On the contrary, the “Leccese” class samples could be observed at positive values of the t1 component, very discriminated from the other classes. The “Otranto” class appeared separated from the others along the positive values of the t2 component and at close to 0 values for the first component. The loading scatter plot for the model (Figure 5b) revealed an interesting relatively higher content of hydroxycinnamates derivatives for “Leccese” and “Otranto” class samples. In particular, as confirmed by the x variable trend plot for the discriminating variables (Figure 6), the “Leccese” samples showed higher content of cichoric and monocaffeoyl tartaric acids with respect to the other here studied cultivars. In recent years, many studies reported diverse bioactivity of compounds isolated from the chicory; phenolic compounds as chicoric acid, chlorogenic acid, caftaric acid, [62,63], flavonoids [64], inulin [19], sesquiterpene lactones [65], and coumarins [64] were considered as responsible for positive impact on human health. In particular, chicoric acid is the main compound in *C. intybus*, as previously found in other studies [55,66]. Moreover, *C. intybus* leaves are specifically rich in chicoric acid as reported [63,67]. Chicoric acid, a caffeic acid derivative, has been also regarded as a nutraceutical with antioxidant and anti-inflammatory activities [68,69] as well as antioxidant effects, important for human health [70]. In addition to anti-inflammation [69,71,72] and antioxidation effects [73,74], the main benefits of chicoric acid also include antiviral [75,76,77], glucose and lipid homeostasis [78,79] and neuroprotection [80] activities. The above reported multi bioactivities of chicoric acid clearly suggest that it has a great potential related to health benefits.

Finally, we performed two pair wise OPLS-DA analysis between “Galatina” and “Bianca” and also between “Leccese” and “Otranto” varieties by considering the aromatic region, with the aim to highlight differences between these varieties. On the other hand, the possible presence of low-intensity signals of potential discriminating metabolites could be evaluated.

**Figure 7:**
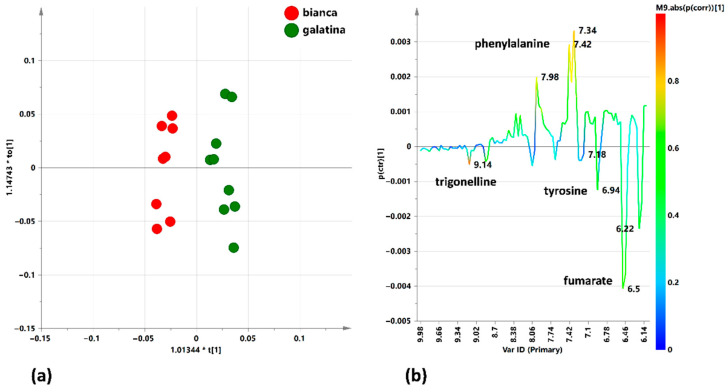
(**a**) Orthogonal Partial Least Squares Discriminant Analyses (OPLS-DA) t[1]/to [1] scores plot for “Bianca” and “Galatina” local varieties (1 + 1 + 0, R^2^X = 0.671, R^2^Y = 0.941, Q^2^ = 0.842) focusing aromatic spectral region. (**b**) S-line plot for the model, colored according to the correlation scaled coefficient (* *p*(corr) ≥ |0.5|). The colour bar associated to the plot indicates the correlation of the metabolites in segregating among classes.

In the “Galatina” vs. “Bianca” comparison, the resulting model was characterized by a clear separation between the two considered classes and described by good descriptive and predictive parameters (1 + 1 + 0, R^2^X = 0.671, R^2^Y = 0.941, Q^2^ = 0.842) (Figure 7a). The S line plot for the model indicated the presence of higher relative content of phenylalanine for “Galatina” varieties (Figure 7b). On the contrary, a higher relative content of tyrosine, fumarate and trigonelline, an important alkaloid already described for its therapeutic properties [81] was observed in the Bianca class.

**Figure 8:**
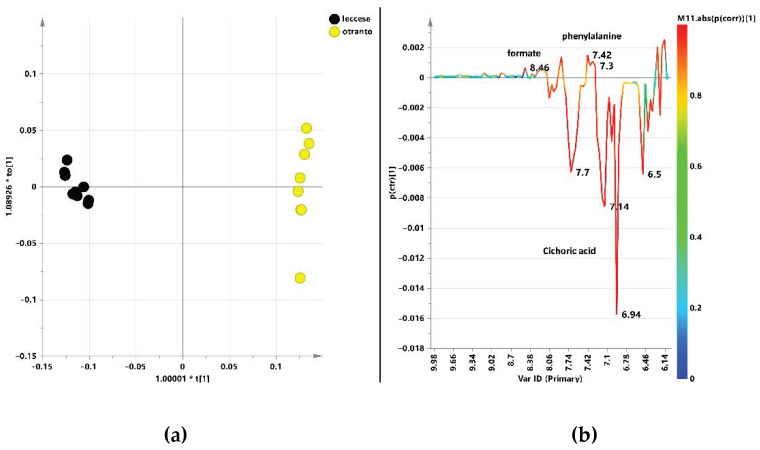
(**a**) OPLS-DA t[1]/to [1] scores plot for “Otranto”and “Leccese” local varieties (1 + 1 + 0, R^2^X = 0.948, R^2^Y = 0.996, Q^2^ = 0.995) focusing aromatic spectral region). (**b**) S. line plot for the model, colored according to the correlation scaled coefficient (* *p*(corr) ≥ |0.5|). The colour bar associated to the plot indicates the correlation of the metabolites in segregating among classes.

Another pair wise OPLS-DA analysis was then performed by comparing the metabolic profiles of “Leccese” and “Otranto” in the aromatic region. Moreover, in this case, the resulting OPLS-DA model (Figure 8a) showed a clear separation between the two considered classes with excellent descriptive and predictive parameters (1 + 1 + 0; R^2^X = 0.948, R^2^Y = 0.996; Q^2^ = 0.995). The S line plot for the model indicated the presence of higher relative content of aromatic amino acid phenylalanine and formate for “Otranto” varieties (Figure 8b). On the contrary, a strong higher content of cichoric acid was discriminating for “Leccese” varieties. Moreover, cichoric acid (6.94 ppm, 7.7 ppm, and 6.5 ppm) could be considered as a reliable marker for the considered class with high pcorr values as indicated by the red color in the line plot.

### 3.3. Quantitative Metabolites Comparison

**Figure 9:**
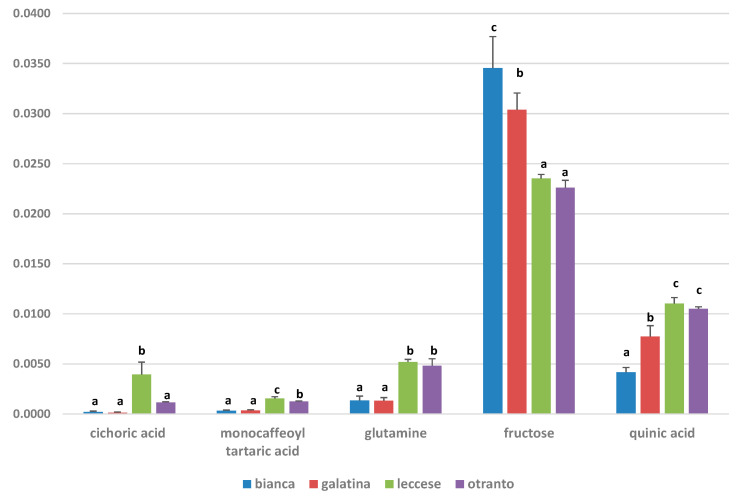
Discriminant metabolites multiple comparison graphical summary for “Bianca”, “Galatina”, “Leccese”, and “Otranto” chicory varieties. The bar plots show the original values (mean +/− standard deviation of integrals corresponding to specific Nuclear Magnetic Resonance (NMR) peak related buckets for each group). Values with different letters indicate significant differences of metabolite level. (Multiple Comparisons of Means test Tukey’s honestly significant difference (HSD) post hoc test).

The variation in discriminating metabolites content for the different local varieties of *C. intybus* was also determined by quantitative comparison of the buckets corresponding to selected distinctive unbiased NMR signals (Figure 9). In particular, relative quantification based on the mean of the buckets corresponding to quinic (binned at 2.06 ppm), cichoric acid (binned at 6.94 ppm), monocaffeoyl tartaric acid (6.86 ppm), fructose (binned at 4.10 ppm) and glutamine (2.42 ppm) signals were considered. Metabolites that showed a significant variation among groups were validated by one-way ANOVA with the HSD post-hoc test and are reported as the mean and standard deviation of integrals corresponding to specific NMR peak related buckets for each group (Figure 9 and Table S2). A significant higher content of monocaffeoyl tartaric and quinic acids together with glutamine, was observed for “Leccese” and “Otranto” with respect both “Galatina” and “Bianca” varieties. For cichoric acid a significant variation was observe among “Leccese” and both “Galatina” and “Bianca” varieties, with a relatively higher content for the “Leccese”. On the other hand, “Bianca” and “Galatina” varieties showed a significant higher content of fructose with respect to both “Leccese” and “Otranto” varieties. The Log2 fold change (FC) ratios were also reported (Figure S3).

## 4. Conclusions

The here reported characterization of *C. intybus* local varieties, by ^1^H NMR based metabolomics, allowed to determine their potential usefulness as well as to define specific qualitative-nutritional characteristics. The obtained results could improve the knowledge on these local varieties also highlighting the presence of compounds with nutraceutical activities such as chicoric acid. Further investigations are required to fully achieve a complete characterization of the studied chicory varieties. Nevertheless the acquisition of preliminary data suitable for a possible origin certification for these local products could be considered as a further outcome of this study.

## Figures and Tables

**Table 1:** ^1^H- chemical shifts of assigned metabolites. Signals refers to TSP signals (0 ppm).

Metabolites	Chemical Shifts δ (ppm)
**Aminoacids**	
Leucine	0.97 (d, β-CH_3_), 1.72 (d, β-CH_2_)
Valine	0.98 (d, CH_3_), 1.03 (d, CH_3_), 2.26 (m, β-CH)
Isoleucine	1.00 (d, β−CH_3_)
Threonine	1.32 (d, γ-CH_3_), 4.26 (α-CH)
Alanine	1.47 (d, CH_3_), 3.79 (m, α-CH)
GABA (γ-aminobutyric acid)	1.90 (m, β-CH_2_), 2.35 (t, α-CH_2_), 3.02 (t, γ-CH_2_)
Glutamine	2.13 (m, β-CH_2_), 2.44 (m, γ-CH_2_), 3.76 (m, α-CH)
Glutamate	2.36 (m, γ-CH_2_)
Asparagine	2.95 (dd, β-CH_2_)
Tyrosine	6.91 (m, CH3, H5), 7.19 (m, CH2, CH6)
Phenylalanine	7.43 (m, CH-3, 5, ring), 7.37 (m, CH-4, ring), 7.30 (m, CH-2,6)
**Sugars**	
β-D-glucose	3.26 (dd, CH-2), 3.48 (t, CH-3),4.64 (d, CH-1)
α-D-glucose	3.5 (dd, H2), 5.23 (d, H1)
Sucrose	3.55 (dd, CH-2), 3.67 (s, CH-2′), 3.81 (m, CH_2_-6,6′), 4.20 (d, H3′), 5.40 (d, CH-1)
α-D-fructofuranose	4.01 (CH-5), 4.1 (d, CH-3,)
β-D-fructofuranose	4.12 (m, CH-3, CH-4), 3.80 (m, CH-5)
β-D-fructopyranose	4.02 (CH-5), 3.70, 3.56 (CH_2_-1,1′)
Inulin	5.42 (m, CH-1), 4.28 (m, CH-3′)
**Organic acids**	
Malate	2.39 (β-CH), 2.69 (β’-CH), 4.31 (α-CH)
Tartrate	4.31(s, CH)
Fumarate	6.52 (α, β-CH=CH)
Formate	8.46 (s, HCOOH)
**Phenolic compounds**	
Cichoric acid	5.54 (s, CH(O)COOH), 6.50 (d, =CH-COO^−^), 6.97 (d, CH5′), 7.26 (d, CH-2′), 7.72 (d, -CH=)
Monocaffeoyl tartaric acid	6.89 (d, CH-5′), 7.62 (d, -CH=), 6.43 (d, =CH-COO^−^), 5.30 (d, CH(O)COOH)
Chlorogenic acid	7.61 (d, -CH=), 6.35 (d, =CH-COO^−^), 5.32 (d, CH(O)COOH)
**Other compounds**	
fatty acids	0.9
uridine	7.9 (d, CH-5, ring), 5.9 (CH-6, ring)
deoxyadenosine	8.3 (s, CH-13 ring), 8.2(s, CH-11, ring)
trigonelline	9.13 (s, CH-2), 8,84 (t, CH-3,5)

## Data Availability

Data is contained within the article and in the supplementary material.

## Supplementary Materials

The following are available online at https://www.mdpi.com/article/10.3390/ijerph18084057/s1, Figure S1: Detail of ^1^H-^13^C hsqc spectrum of chicory aqueous sample. Table S1: Summary of morphological characters scored on 36 chicory samples. Figure S2: DModX plot for the model of Figure 3. The distance to the model of X (DModX), normalized in units of standard deviation. DModX larger than the critical limit indicates that the observation is an outlier in the X space. Figure S3: Discriminating metabolites comparison among local C. intybus varieties provided as values of—Log2 (FC). Metabolites with—Log2 (FC) negative values have higher concentration in “Otranto” (O) and “Leccese” (L) varieties, while—Log2 (FC) positive values indicated metabolites with higher concentration in “Bianca“ (B) and “Galatina” (G) varieties. (Multiple Comparisons of Means test Tukey’s honestly significant difference (HSD) post hoc test). Statistical significance, indicated with *, was set at least at an adjusted *p*-values < 0.05. Table S2: Quantitative comparison of *C. intybus* local varieties discriminant metabolites.
