# Supplementary material for: First Insight into Nutraceutical Properties of Local Salento Cichorium intybus Varieties: NMR-Based Metabolomic Approach
Source: Int J Environ Res Public Health. 2021 Apr 12;18(8):4057. doi: 10.3390/ijerph18084057 (PMC8069254; doi:10.3390/ijerph18084057)
Supplement: Supplementary file 1 [file ijerph-18-04057-s001.pdf]

Supplementary Material

First insight into nutraceutical properties of local Salento *Cichorium intybus* varieties: NMR-based metabolomic approach

Chiara Roberta Girelli, Francesca Serio, Rita Accogli, Federica Angilè , Antonella De Donno and Francesco Paolo Fanizzi*

[1] Department of Biological and Environmental Sciences and Technologies, University of Salento, Prov.le Lecce-Monteroni, 73100 Lecce, Italy; chiara.girelli@unisalento.it (C.R.G.); francesca.serio@unisalento.it (F.S.); rita.accogli@unisalento.it (R.A.); federica.angile@unisalento.it (F.A.); antonella.dedonno@unisalento.it (A.D.D.); fp.fanizzi@unisalento.it (F.P.F.)

* fp.fanizzi@unisalento.it; Tel.: +39-0832-29265

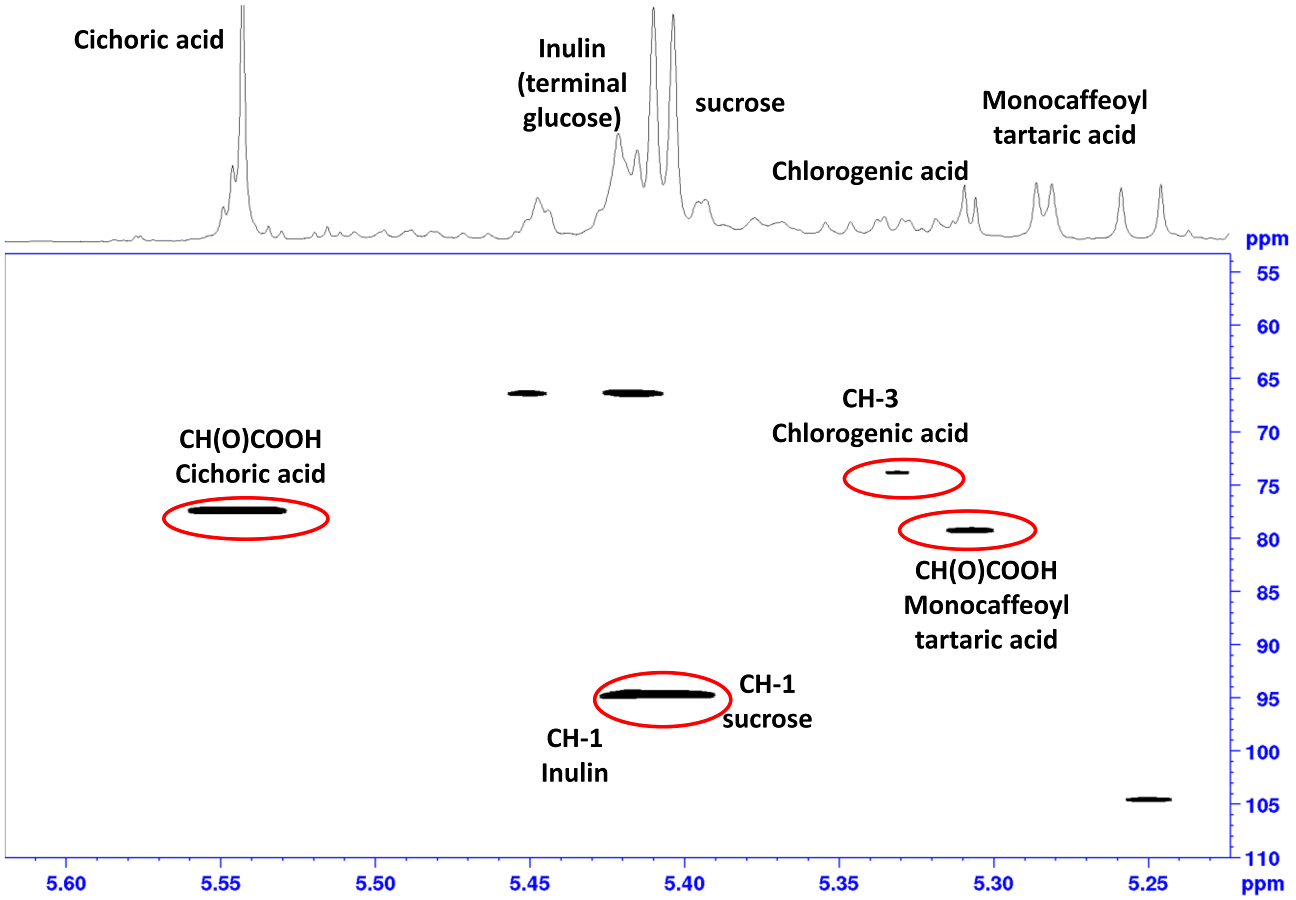

Figure S1. Detail of ^{1}H-^{13}C hsqc spectrum of chicory aqueous sample.

Table S1. Summary of morphological and physiological characters scored on 36 chicory samples.

**Chicory variety**	**“Bianca”**	**“Galatina”**	**“Leccese”**	**“Otranto”**
Origin	Tricase 39° 55' 48 N; 18° 21' 15 E	Galatina 40°10'30''00 N; 18°10'8''40 E	Lecce 40°21'28''80 N; 18°10'22''08 E	Otranto 40°8'55''68 N; 18°29'12''12 E
**Cultivation**				
Harvest time	nov-feb	feb-apr	may-july	may-sept
Crop cycle duration	6 months	6 months	3-5 months	6 months
Commercial ripening stage	early	early	late	late
**Edible part**				
Edible part height	51,52±5.32 cm	51,9±5,7 cm	66,6±7,89 cm	51,6±13,7 cm
Bud size	4-10 cm	3-6 cm	10-30 cm	10-30 cm
Bud weight			20-25 g	20-25 g
Head weight	400-1000 g	350-450 g		
Plant height	51,91±5,69 cm	51,97±4,84 cm	70,1±7,5 cm	55,2±13,44 cm
Plant diameter	14,26 ± 1,91 cm	15,55±1,67 cm	17,2± 1,5 cm	19,62± 2,6 cm
Number of shoots	14,67± 3,82	25,33± 9,4	11,3±3,1	13,8±4,1
**Leaf**				
Length	48,80 ± 5,55 cm	56,55±4,72 cm	31,4±3,88 cm	33,9±5,2 cm
Width	7,58 ± 2.7 cm	11,99±3,27 cm	7,72±2,36 cm	9,3±3,91 cm
Length / width relationship	8,18±2,44 cm	5,45±3,75 cm	4,28±16,75 cm	4,5±16,26 cm
Main leaf rib	2,82±0,38 cm	3,56±0,23 cm	1,34±0,41 cm	2,6±0,62 cm
Shape	elliptical	elliptical	elliptical	elliptical
Color	green	green	green	green
Color intensity	light	light	light	light
Brilliance	weak	medium	weak	medium
Main leaf rib color	white	white	white	white
Vertical profile of leaf	weakly concave	weakly concave	weakly concave	weakly concave
Blistering	strong	medium	weak	weak
Undulation	medium	very strong	very strong	very strong

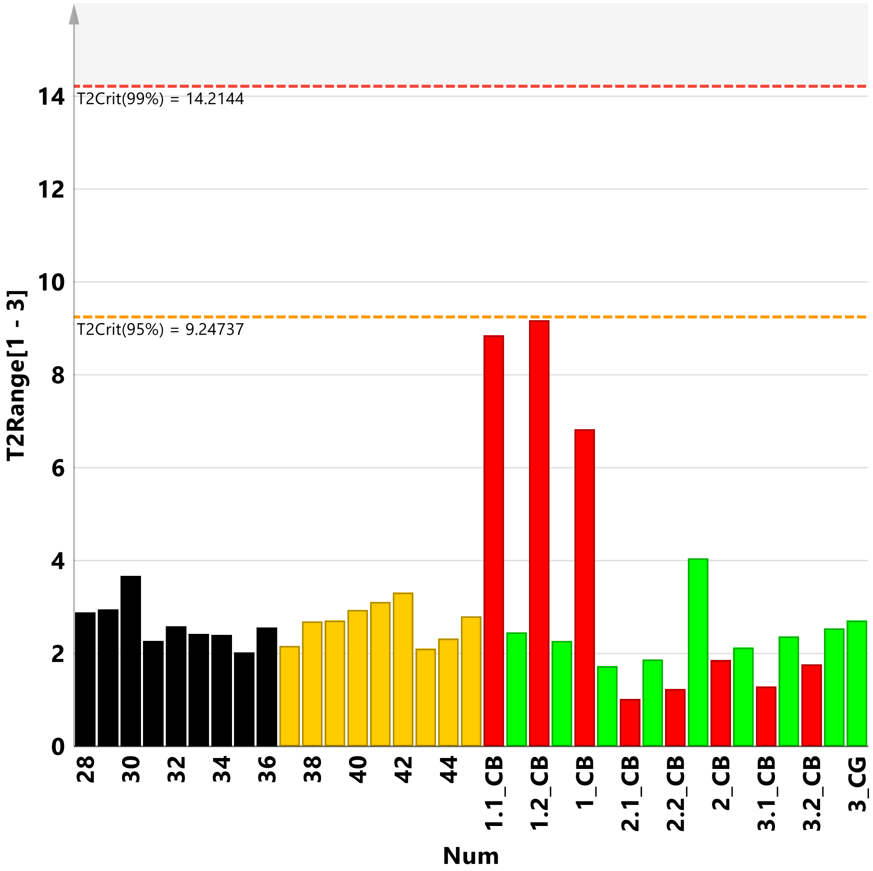

Figure S2. DModX plot for the model of Figure 3. The distance to the model of X (DModX), normalized in units of standard deviation. DModX larger than the critical limit indicates that the observation is an outlier in the X space.

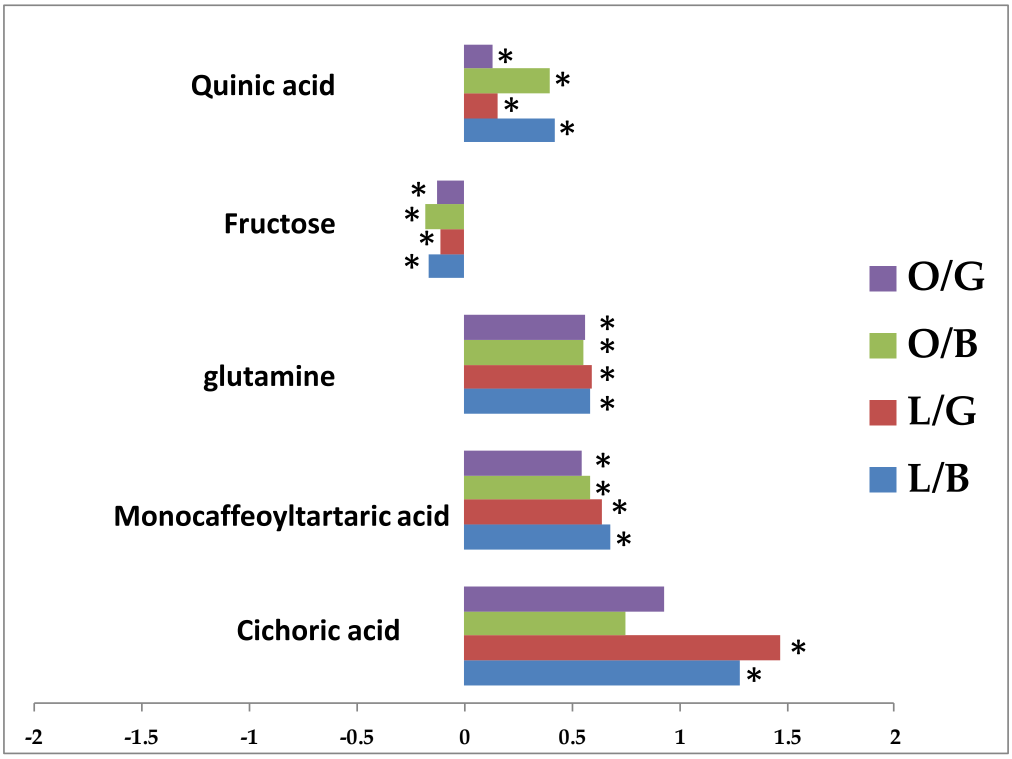

Figure S3. Discriminating metabolites comparison among local *C. intybus* varieties provided as values of − Log2 (FC). Metabolites with − Log2 (FC) negative values have higher concentration in "Otranto" (O) and "Leccese" (L) varieties, while − Log2 (FC) positive values indicated metabolites with higher concentration in "Bianca" (B) and "Galatina" (G) varieties. (Multiple Comparisons of Means test Tukey's honestly significant difference (HSD) post hoc test). Statistical significance, indicated with *, was set at least at an adjusted p-values < 0.05.

Table S2. Quantitative comparison of *C. intybus* local varieties discriminant metabolites

	**Mean ± S.D.**[1]							
**Metabolite(ppm)**	**B**	**G**	**L**	**O**	**f value**[2]	**FDR**[3]	**p-value**[4]	**Tukey's HSD**[5]
Quinic acid (2.06)	$4.2x10^{-3}±0.5x10^{-3}$	$7.7x10^{-3}$ $±1.1x10^{-3}$	$11.0x10^{-3}±0.6x10^{-3}$	$10.5x10^{-3}$ $±0.2x10^{-3}$	182.08	$1.53x10^{-19}$	$5.39x10^{-21}$	G-B; L-B; O-B; L-G; O-G;
Fructose (4.10)	$34.5\ x10^{-3}$ $±3.1\ x10^{-3}$	$30.4x10^{-3}±1.7x10^{-3}$	$23.5x10^{-3}$ $±0.4x10^{-3}$	$22.6x10^{-3}$ ±0.7	90.633	$2.45x10^{-15}$	$1.96x10^{-15}$	B-G; B-L; B-O; G-L; G-O;
Glutamine (4.34)	$1.3x10^{-3}$ $±0.4x10^{-3}$	$1.3x10^{-3}±0.3x10^{-3}$	$5.2x10^{-3}±0.3x10^{-3}$	$4.8x10^{-3}$ $±0.7x10^{-3}$	201.9	$5.02x10^{-20}$	$2.01x10^{-20}$	L-B; O-B; L-G; O-G;L-O;
Monocaffeoyl tartaric acid (6.86)	$0.3x10^{-3}±0.1x10^{-3}$	$0.4x10^{-3}±0.1$	$1.5x10^{-3}$ $±0.2\ x10^{-3}$	$1.2x10^{-3}$ $±0.1x10^{-3}$	257.8	$2.69x10^{-21}$	$5.39x10^{-21}$	L-B; O-B; L-G; O-G;L-O;
Cichoric acid (6.94)	$0.2x10^{-3}$ $±0.1x10^{-3}$	$0.1x10^{-3}$ $±0.01x10^{-3}$	$3.9x10^{-3}$ $±1.2x10^{-3}$	$1.2x10^{-3}$ $±0.1x10^{-3}$	73.348	$3.56x10^{-14}$	$3.56*10^{-14}$	L-B; L-G; L-O;

[1] Mean and relative standard deviation refers to the relative buckets of metabolite corresponding NMR signal, determined from 1D ^{1}H-NMR spectra of each classes. [2] F value = Variance of the group means (Mean Square Between)/mean of the within group variances (Mean Squared Error); [3] False Discovery Rate (FDR); [4] Statistical significance was set at p-value < 0.05 with the 95% confidence level; [5] Tukey's Honestly Significant Difference (HSD) post hoc test. Adjusted p-value (FDR) cutoff: 0.05. Letters indicate the four varieties: Bianca (B); Galatina (G); Leccese (L); Otranto (O).
